# Revisiting Endoreversible Carnot Engine: Extending the Yvon Engine

**DOI:** 10.3390/e27020195

**Published:** 2025-02-13

**Authors:** Xiu-Hua Zhao, Yu-Han Ma

**Affiliations:** 1 School of Physics and Astronomy, Beijing Normal University, Beijing 100875, China; 2 Key Laboratory of Multiscale Spin Physics (Ministry of Education), Beijing Normal University, Beijing 100875, China; 3 Graduate School of China Academy of Engineering Physics, Beijing 100193, China

**Keywords:** finite-time thermodynamics, endoreversible heat engine, Curzon–Ahlborn efficiency, Yvon engine

## Abstract

Curzon and Ahlborn’s 1975 paper, a pioneering work that inspired the birth of the field of finite-time thermodynamics, unveiled the efficiency at maximum power (EMP) of the endoreversible Carnot heat engine, now commonly referred to as the Curzon–Ahlborn (CA) engine. Historically, despite the significance of the CA engine, similar findings had emerged at an earlier time, such as the Yvon engine proposed by J. Yvon in 1955 that shares the exact same EMP, that is, the CA efficiency $\eta_{\mathrm{CA}}$. However, the special setup of the Yvon engine has circumscribed its broader influence. This paper extends the Yvon engine model to achieve a level of generality comparable to that of the CA engine. With the power expression of the extended Yvon engine, we directly explain the universality that $\eta_{\mathrm{CA}}$ is independent of the heat transfer coefficients between the working substance and the heat reservoirs. A rigorous comparison reveals that the extended Yvon engine and CA engine represent the steady-state and cyclic forms of the endoreversible Carnot heat engine, respectively, and are equivalent.

## 1. Introduction

The seminal paper by Curzon and Ahlborn [1], published in 1975, is a landmark work in finite-time thermodynamics [2]. By analyzing an endoreversible Carnot engine with finite temperature differences between the working substance and the reservoirs, the authors optimized the power output of the engine and obtained the efficiency at maximum power (EMP), which is a more practical efficiency bound than the Carnot efficiency [3]. The EMP formula they derived,

(1) $$\eta_{\mathrm{CA}}=1-\sqrt{T_{c}/T_{h}}$$

with $T_{c}$ ($T_{h}$) being the temperature of the cold (hot) reservoir, is commonly referred to as Curzon–Ahlborn (CA) efficiency. In the following decades, studies on finite-time heat engines have advanced rapidly [2,4,5,6,7,8]. Notable topics include the nature of CA efficiency [9,10,11] and the microscopic realization of CA engines [12,13], various forms of endoreversible thermodynamic cycles [14,15,16,17], different theoretical frameworks for finite-time cycles [10,18,19,20,21,22] and their thermodynamic bounds [15,23,24,25,26,27], and the connections and shared principles among diverse heat engine models [28,29,30].

In fact, Curzon and Ahlborn’s work was not the first to explore the performance of finite-time heat engines or derive the CA efficiency [31,32,33,34]. As early as 1955, Yvon presented the same formula as Equation (1) while investigating the optimization of actual power plants modeled as endoreversible engines [35]. Two years later, Chambadal [36] and Novikov [37] presented discussions that are quite similar to that of Yvon and reached the same conclusion. Yvon’s approach, though yielding the same EMP expression, differed from Curzon and Ahlborn’s in two key aspects as follows: (i) a finite temperature difference was assumed only between the working substance and the hot reservoir, with no temperature difference on the cold side; and (ii) instead of explicitly considering cycle time, finite heat flux and work rate were directly used to characterize the finite-time nature of practical engines. These distinctions render the relationship between the Yvon and CA engines less straightforward, obfuscating the grounds for their having the same EMP.

The simplicity of Yvon’s optimization procedure (shown in Section 2) inspires us to extend their model to encompass more general finite-time engines. The extended Yvon engine presented in Section 3 provides a unified and straightforward framework for studying endoreversible Carnot engines, bridging the gap between Yvon’s original analysis and the broader scope of Curzon and Ahlborn’s work. Specifically, we relax the assumption of equal temperatures on the cold side in the Yvon engine, and optimize the work rate (power) with respect to the endoreversible temperatures of the working substance under the endoreversible condition. The derived EMP aligns with the CA efficiency, yet the optimization process is significantly more concise than that of the CA engine. Moreover, our approach naturally reproduces the trade-off relation between power and efficiency beyond the maximum-power regime [15], a topic of considerable interest in contemporary thermodynamic research [8,38]. In Section 4, we provide a rigorous comparison between the extended Yvon engine and the CA engine models to demonstrate their equivalence in describing the endoreversible Carnot heat engine. The conclusion, outlook, and some remarks of this study are presented in Section 5.

## 2. Yvon Engine

**Figure 1:**
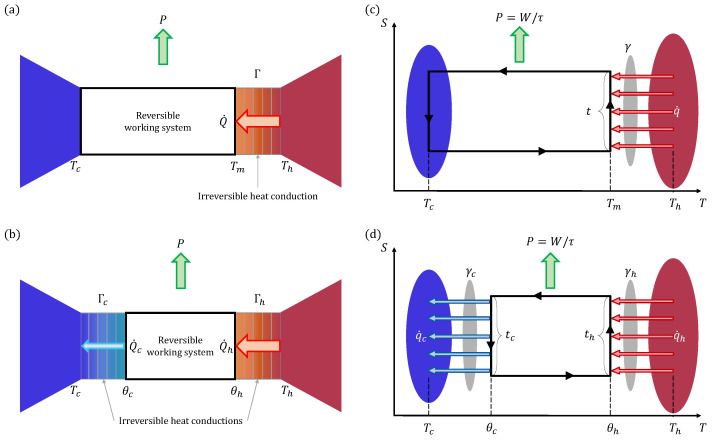
Steady-state (**a**,**b**) and cyclic (**c**,**d**) endoreversible heat engines. (**a**) In the original Yvon engine, finite heat flux (denoted as $\dot{Q}$) occurs only at the high-temperature end, where there is a temperature difference between the working substance and the hot reservoir. (**b**) The extended Yvon engine introduces temperature differences, and thus, heat fluxes, between the working substance and both the hot and cold reservoirs. (**c**,**d**) show the entropy (*S*)–temperature (*T*) diagrams of the endoreversible Carnot engine cycles with finite heat fluxes (denoted as $\dot{q}$) along the high-temperature and both isothermal branches, respectively.

We start with reviewing Yvon’s pioneering treatment of the finite-time Carnot heat engine [33,35]. Yvon’s report [35] at the 1955 United Nations Conference on the Peaceful Uses of Atomic Energy offered a simple solution for maximizing the output power of the steam engine in nuclear reactors. Specifically, as shown in Figure 1a, the fluid at temperature $T_{h}$ from the reactor core serves as the hot reservoir transferring heat to the hot end of the working substance in the engine, where the temperature of this end is maintained at $T_{m}<T_{h}$. The Yvon engine generates power through the turbine shafts and isothermally releases heat to the condenser where the working substance and the condenser are at the same lowest temperature $T_{c}<T_{m}$. This assumes that the heat flux from the fluid to the working substance obeys Newton’s law of cooling, namely,

(2) $$\dot{Q}=\Gamma \left(T_{h}-T_{m}\right),$$

where Γ is a constant that depends on the thermal conductivity and area of the wall separating the working substance from the hot fluid, and the overdot notation stands for the rates. This heat transfer law, proportional to the temperature difference between the reservoir and the working substance, is valid for small temperature differences [39,40,41], which is the scope of our study. The heat engine efficiency is defined as $\eta \equiv P/\dot{Q}$, where *P* is the output power of the engine. With the assumption that the working substance undergoes a reversible transformation between $T_{m}$ and $T_{c}$, namely, the heat engine is equivalent to operating with a Carnot cycle between a hot reservoir of temperature $T_{m}$ and a cold reservoir of temperature $T_{c}$, the efficiency of the engine reads as

(3) $$\eta^{\left(Y\right)}=1-T_{c}/T_{m},$$

which is now known as the endoreversible assumption [42]. Hence, the power of the Yvon engine follows as

(4) $$P^{\left(Y\right)}=\eta^{\left(Y\right)}\dot{Q}=\Gamma \left(T_{h}+T_{c}-T_{m}-\frac{T_{h}T_{c}}{T_{m}}\right)\leq \Gamma {\left(\sqrt{T_{h}}-\sqrt{T_{c}}\right)}^{2},$$

where the AM-GM inequality, $\left(a+b\right)/2\geq \sqrt{ab}$ for nonnegative *a* and *b*, has been applied. The power output is optimized to determine a practical operating regime for real engines, where high power is required to perform real-world tasks. Associated with the third equality in Equation (4), the maximum power is achieved when

(5) $$T_{m}=T_{m}^{*}\equiv \sqrt{T_{h}T_{c}},$$

and the corresponding efficiency at maximum power $\eta^{\left(Y\right)}{|}_{T_{m}=T_{m}^{*}}=\eta_{\mathrm{CA}}$ is easily checked.

## 3. Extended Yvon Engine

In the Yvon engine, heat flux is only considered when the working substance absorbs heat from the hot reservoir. In this section, we extend the model to include heat flux on the cold side, resulting from the temperature difference between the working substance and the cold reservoir, as illustrated in Figure 1b. This extended Yvon engine is consistent with the CA engine [Figure 1d], though it uses a different representation method based on energy fluxes rather than examining each branch of a thermodynamic cycle.

The heat flux from the hot reservoir at temperature $T_{h}$ to the working substance at temperature $\theta_{h}<T_{h}$ follows

(6) $${\dot{Q}}_{h}=\Gamma_{h}\left(T_{h}-\theta_{h}\right),$$

where the constant $\Gamma_{h}$ characterizes the heat conductivity during heat absorption. Similarly, the heat flux from the working substance, whose temperature decreases to $\theta_{c}<\theta_{h}$, to the cold reservoir at temperature $T_{c}<\theta_{c}$ is

(7) $${\dot{Q}}_{c}=\Gamma_{c}\left(\theta_{c}-T_{c}\right),$$

with $\Gamma_{c}$ being a constant during heat release. As a result of the energy conservation law, the engine’s output power is

(8) $$P={\dot{Q}}_{h}-{\dot{Q}}_{c}=\eta {\dot{Q}}_{h}.$$

In this case, the endoreversible assumption reads as

(9) $$\frac{{\dot{Q}}_{h}}{\theta_{h}}=\frac{{\dot{Q}}_{c}}{\theta_{c}},$$

which indicates that there is no entropy generation in the working substance for this steady-state engine, and the entropy flow from the hot reservoir to the working substance is balanced by the entropy flow from the working substance to the cold reservoir [43]. Correspondingly, $\eta =1-\theta_{c}/\theta_{h}$ is the endoreversible efficiency. According to Equations (6), (7), and (9), $\theta_{h}$ can be expressed with the temperature ratio $\theta_{h}/\theta_{c}$ as

(10) $$\theta_{h}=\frac{\Gamma_{h}T_{h}}{\Gamma_{h}+\Gamma_{c}}+\frac{\Gamma_{c}T_{c}}{\Gamma_{h}+\Gamma_{c}}\frac{\theta_{h}}{\theta_{c}},$$

substituting which into Equation (8), we obtain

(11) $$P=\frac{\Gamma_{h}\Gamma_{c}T_{h}}{\Gamma_{h}+\Gamma_{c}}\left(1+\frac{T_{c}}{T_{h}}-\frac{\theta_{c}}{\theta_{h}}-\frac{T_{c}}{T_{h}}\frac{\theta_{h}}{\theta_{c}}\right).$$

Similar to Equation (4), by utilizing the AM-GM inequality, it is easy to find that, for given $\Gamma_{h,c}$ and $T_{h,c}$,

(12) $$P\leq \frac{\Gamma_{h}\Gamma_{c}T_{h}}{\Gamma_{h}+\Gamma_{c}}{\left(1-\sqrt{\frac{T_{c}}{T_{h}}}\right)}^{2}\equiv P_{\max},$$

where the maximum power $P_{\max}$ is achieved with the optimal endoreversible temperatures $\theta_{h}^{*}$ and $\theta_{c}^{*}$, which satisfy

(13) $$\frac{\theta_{c}^{*}}{\theta_{h}^{*}}=\sqrt{\frac{T_{c}}{T_{h}}}.$$

Consequently, the EMP of the engine $\eta_{\mathrm{MP}}=1-\theta_{c}^{*}/\theta_{h}^{*}=\eta_{\mathrm{CA}}$. In the limiting case of $\Gamma_{h}/\Gamma_{c}\to 0$, it follows from Equations (10) and (13) that

(14) $$\theta_{h}^{*}=\sqrt{T_{h}T_{c}}=T_{m}^{*},\;\;\theta_{c}^{*}=T_{c},$$

and the original Yvon engine is recovered. With nonvanishing $\Gamma_{h}/\Gamma_{c}$, the maximum power of the extended Yvon engine [Equation (12)] is lower than that of the original Yvon engine [Equation (4)], despite operating at the same efficiency, due to the additional dissipation resulting from the finite-rate heat transfer at the low-temperature end.

**Figure 2:**
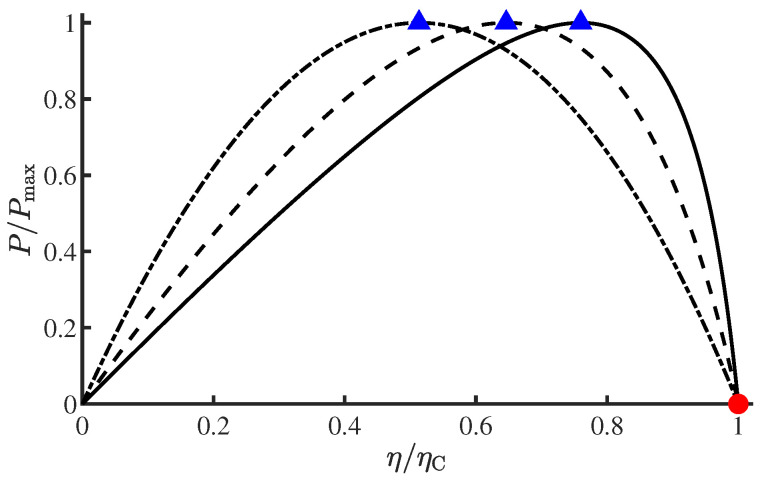
Trade-off relations between power and efficiency for the Curzon-Ahlborn engine with $\eta_{C}=0.1$ (dash-dotted curve), $\eta_{C}=0.7$ (dashed curve), and $\eta_{C}=0.9$ (solid curve). The triangles and circle mark the maximum power and maximum efficiency, respectively.

Furthermore, in Equation (11), replacing the temperature ratios with efficiencies, where $T_{c}/T_{h}=1-\eta_{C}$ and $\theta_{c}/\theta_{h}=1-\eta$, yields

(15) $$P=\frac{T_{h}}{\Gamma_{h}^{-1}+\Gamma_{c}^{-1}}\frac{\eta \left(\eta_{C}-\eta \right)}{\left(1-\eta \right)},$$

which is illustrated in Figure 2 for $\eta_{C}=0.1,\;0.7,\;0.9$ with normalized axes. This relation determines the efficiency at an arbitrary power or the power at an arbitrary efficiency of the endoreversible Carnot engine. The explicit dependence of power on efficiency, which provides a comprehensive optimization regime for thermal machines, is now referred to as the trade-off or constraint relation between power and efficiency [23,24,25,44,45], and this constitutes one of the key focuses within the realm of finite-time thermodynamics [30]. For more details and the related progress on this issue, please refer to Ref. [8] along with the references encompassed therein.

## 4. Comparison of Curzon–Ahlborn and Extended Yvon Engines

Both the Yvon engine and its extended version are steady-state heat engines, while the CA engine adopts a cyclic representation, taking into account the duration of each thermodynamic process in the cycle. Although the previous section has demonstrated the consistency of the EMP obtained by the extended Yvon and CA engines, their strict correspondence needs further clarification. In this section, we will establish the strict correspondence between the parameters of the two models. As the cyclic counterpart of the extended Yvon engine [Figure 1b], the CA engine is depicted in Figure 1d in the entropy–temperature (S−T) diagram. Meanwhile, Figure 1c represents the limiting case of the CA heat engine, where the temperature difference at the low-temperature end approaches zero, corresponding to the original Yvon engine illustrated in Figure 1a.

In Curzon and Ahlborn’s original derivation [1], engine power is expressed not through energy fluxes but as the total work $W=Q_{h}-Q_{c}$ divided by the duration $\tau =\xi (t_{h}+t_{c})$ of an endoreversible Carnot cycle, namely,

(16) $$P^{\left(\mathrm{CA}\right)}=\frac{Q_{h}-Q_{c}}{\xi \left(t_{h}+t_{c}\right)}.$$

Here, $t_{h}$ and $t_{c}$ represent the durations of the heat absorption and release processes [see Figure 1d], respectively, while the time taken to complete the adiabatic transitions, $\left(\xi -1\right)\left(t_{h}+t_{c}\right)$, is assumed to be proportional to the duration of the isothermal processes with ξ>1. The exchanged heat, $Q_{h(c)}=\int {\dot{q}}_{h(c)}dt$, in the isothermal processes is

(17) $$Q_{h}=\gamma_{h}\left(T_{h}-\theta_{h}\right)t_{h},\;Q_{c}=\gamma_{c}\left(\theta_{c}-T_{c}\right)t_{c},$$

where $T_{h,c}$ and $\theta_{h,c}$ have the same meanings as in the extended Yvon model. However, the heat transfer coefficients $\gamma_{h,c}$ are different from $\Gamma_{h,c}$, as will be discussed later. As the result of the endoreversible assumption and the cyclic condition, the entropy variation of the working substance in a cycle satisfies

(18) $$\Delta S=\frac{Q_{h}}{\theta_{h}}-\frac{Q_{c}}{\theta_{c}}=0.$$

Consequently, this cyclic engine’s efficiency $\eta \equiv \left(Q_{h}-Q_{c}\right)/Q_{h}=1-\theta_{c}/\theta_{h}$. Comparing the first equality in Equation (8) with Equation (16) and Equation (9) with Equation (18), we obtain the relation between $\gamma_{h,c}$ and $\Gamma_{h,c}$ as

(19) $$\Gamma_{h(c)}=\frac{t_{h(c)}}{\xi \left(t_{h}+t_{c}\right)}\gamma_{h(c)}.$$

This equation shows that the heat transfer coefficients in the steady-state engine differ from those in the cyclic engine by a time-proportional factor, which is determined by the ratio of the corresponding process duration to the total cycle duration.

According to Equation (19), the overall coefficient appearing in the upper bound of Equation (12) satisfies

(20) $$\frac{\Gamma_{h}\Gamma_{c}}{\Gamma_{h}+\Gamma_{c}}=\frac{\gamma_{h}\gamma_{c}}{\xi \left(1+t_{c}/t_{h}\right)\left(\gamma_{h}t_{h}/t_{c}+\gamma_{c}\right)}\leq \frac{\gamma_{h}\gamma_{c}}{\xi {\left(\sqrt{\gamma_{h}}+\sqrt{\gamma_{c}}\right)}^{2}},$$

where $\gamma_{h}t_{h}/t_{c}+\gamma_{c}t_{c}/t_{h}\geq 2\sqrt{\gamma_{h}\gamma_{c}}$ has been used, and the equality is saturated with the optimal time ratio

(21) $$\frac{t_{h}^{*}}{t_{c}^{*}}=\sqrt{\frac{\gamma_{c}}{\gamma_{h}}}.$$

This indicates that when the process durations are taken into account, cyclic engines have an additional parameter to be optimized, i.e., process time allocation $t_{c}/t_{h}$, compared to steady-state engines. Substituting Equation (20) into Equation (12), the maximum power of the CA engine [1] is exactly recovered as

(22) $$P_{\max}^{\left(\mathrm{CA}\right)}=\frac{\gamma_{h}\gamma_{c}T_{h}}{\xi {\left(\sqrt{\gamma_{h}}+\sqrt{\gamma_{c}}\right)}^{2}}{\left(1-\sqrt{\frac{T_{c}}{T_{h}}}\right)}^{2}.$$

On the other hand, by combining $\gamma_{h,c}$ and $t_{h,c}$ into $\Gamma_{h,c}$ through Equation (19), the power of the extended Yvon engine given in Equation (11) can be derived from Equations (16)–(18) of the CA engine. In this sense, the extended Yvon engine and the CA engine are equivalent.

Herein, we would like to elucidate why the optimization of the extended Yvon engine exhibits greater succinctness than that of the original CA engine. In the optimization process from Equation (11) to Equation (12), the ratio of $\theta_{c}/\theta_{h}$ emerges as a unified quantity, decoupling its role in maximizing *P* from the heat transfer coefficients $\Gamma_{h,c}$. By contrast, in Ref. [1], the authors individually calculated the derivatives of *P* [Equation (16)] with respect to $\theta_{c}$ and $\theta_{h}$, subsequently determining $\theta_{c}^{*}$ and $\theta_{h}^{*}$ via $\partial P/\partial \theta_{c}=\partial P/\partial \theta_{h}=0$. Eventually, Curzon and Ahlborn arrived at Equation (13) and ascertained its independence from $\Gamma_{h,c}$ or $\gamma_{h,c}$. Essentially, our derivation is more intuitive and effectively highlights the significant universality that the EMP of the endoreversible Carnot engine is invariant to the heat transfer coefficients. Nevertheless, $\Gamma_{h,c}$ impact the specific values of $\theta_{c}^{*}$ and $\theta_{h}^{*}$ via Equations (10) and (13). Furthermore, when correlating heat transfer coefficients in the extended Yvon engine with those of the CA heat engine, Equation (20) indicates that the time ratio $t_{c}/t_{h}$ of different thermodynamic processes in the cyclic heat engine affects the output power as a unified quantity rather than depending on the specific durations of each individual process. As a final point, similarly to the power–efficiency relation Equation (15) for the extended Yvon engine, the trade-off relation between power and efficiency for the CA engine can be obtained by substituting the upper bound in Equation (20) into Equation (15), which was first derived by Chen and Yan [15] through solving ${\left(\partial P/\partial \theta_{h}\right)}_{\eta }=0$. When expressed in normalized form, as illustrated in Figure 2, the power–efficiency trade-offs for both engines are identical.

## 5. Conclusions and Discussion

To summarize, we have extended the original model of the Yvon engine with one-sided heat flux to the general endoreversible Carnot engine with two-sided heat fluxes. The extended Yvon engine and the CA engine are essentially two sides of the same coin, namely the steady-state heat engine form and the cyclic heat engine form of the endoreversible Carnot heat engine. Our derivations emphasize the predominance of the temperature ratio over specific temperature values in optimizing this type of engine, explaining why the EMP of endoreversible engines under Newtonian heat transfer is independent of the heat transfer coefficients. For endoreversible heat engines operating under heat transfer laws other than Newton’s law, the EMP generally depends significantly on the heat transfer coefficients [15].

It is highlighted that the temperature ratio $\theta_{c}/\theta_{h}$ (or efficiency $\eta =1-\theta_{c}/\theta_{h}$) constitutes the sole degree of freedom for steady-state endoreversible engines, independent of the heat transfer coefficients [see Equation (11) or (15)], and decouples from time allocation in cyclic engines [see Equation (20)]. It is worth mentioning that (i) Ref. [46] also recognizes the independent role of efficiency, but only for the special case with symmetric heat transfer coefficients; and (ii) Bejan [31] noticed the fact that different irreversible heat engine models, namely Chambadal’s [36], Novikov’s [37], and Curzon and Ahlborn’s [1] share the same EMP, and he explained this with the theory of entropy generation minimization [47]. Nevertheless, the analyses presented in Ref. [31] did not clarify the strict correspondence between the optimization of cyclic heat engines, which incorporated the process durations as per Curzon and Ahlborn’s methodology, and that of steady-state heat engines characterized by energy fluxes [35,36,37]. We provide such strict correspondence relations in Section 4 to fill this gap.

The optimization approach presented in the current work is intuitive and straightforward, allowing for the direct extension of the optimization criteria, from maximizing power to optimizing efficient power, ecological function, or Omega function [14,48]. The extended Yvon engine has potential applications in areas such as chemical engines [49,50] and thermoelectric generators [51,52]. Future research could further explore the optimal control and geometric optimization [13,53,54,55] of the extended Yvon engine, its performance between finite-sized heat reservoirs [44,56,57,58,59], and its experimental realization [41,45,60]. In addition, the extended Yvon engine can serve as a pedagogical example for teaching thermodynamics and engineering thermodynamics, given that its simple and lucid derivation helps students initiate their understanding of nonequilibrium thermodynamics.

As a final remark, perhaps due to historical, linguistic, or other factors [32,33,34], as well as the particularity of the model, the Yvon engine [35] and its contemporaneous works [36,37] regrettably did not garner the attention they deserved at that time. Twenty years later, the generality and simplicity of the CA engine [1], along with the systematic research on thermodynamics in finite time by the Chicago school [7,61] during the same period, gave birth to the field of finite-time thermodynamics. We hope that the extended Yvon engine proposed in this paper will encourage more people to pay attention to and appreciate Yvon’s ingenious ideas regarding the practical thermodynamic cycle within a finite duration and help disseminate the complete history of finite-time thermodynamics.

## Data Availability

The original contributions presented in this study are included in the article. Further inquiries can be directed to the corresponding author.
